# A Manual of Operative Surgery

**Published:** 1904-06

**Authors:** 


**A Manual of Operative Surgery.** By Sir FREDERICK TREVES, Bart., K.C.V.O., C.B., LL.D. New [2nd] Edition. Revised by the AUTHOR and JONATHAN HUTCHINSON, Jun. Two volumes. London: Cassell & Company Limited. 1903.

Thirteen years have elapsed since the publication of the first edition of this work. During that period very great changes have been made in operative surgery, necessitating the entire revision of the second edition and the re-writing or re-modelling of numerous sections. The most notable alterations occur in the second volume, dealing as it does with the abdomen, genito-urinary system, rectum and thorax.

In carefully reading through this volume the following points indicate the advance made:—

Peritoneal drainage is dismissed in a few words. Rubber drainage-tubes and drains made of sterile gauze are advised when drainage has to be carried out. Sterilised water is alone used for instruments and sponges, the latter being made of sterilised gamgee tissue quilted. The so-called "toilet of the peritoneum" in aseptic cases is strongly condemned.

In the sections on intestinal surgery Murphy's button is described in fullest detail, but is recommended for use only where the saving of the time taken up in suturing is of the utmost importance to the patient, and in operations upon the large bowel. Cushing's right-angled continuous suture is much lauded. Halsted's plain quilt suture is omitted, and Lembert's suture is "still the best form of suture with which we are acquainted." The chapter on intestinal anastomosis has been much curtailed and simplified. Paul's tube is rightly commended for use in the performance of enterotomy. The treatment of intussusception has been much amplified—early abdominal section is urged in acute cases. Barker's operation is recommended in those acute cases where reduction is found to be impossible.

The chapter dealing with the removal of the vermiform appendix has been entirely re-written. "The peritoneum is divided with a scalpel in a circular manner at a point at which the ligature will come. The serous membrane is just sufficiently turned back to make room for the ligature. The turning back of a cuff, or flap, or hood of peritoneum is useless. Such a cuff makes the poorest possible covering for the stump of the appendix, and is probably absolutely bloodless. The practice of applying the actual cautery or pure carbolic acid to the appendix stump is to be condemned. The stump is now sequestrated by a series of Lembert's sutures made of fine silk.

The sutures involve the cæcal wall around the stump of the appendix. The stump is thus inturned and hidden from view, and is perfectly secure."

The chapter on gastro-jejunostomy has also been entirely re-written. The vexed question of the relative superiority of the anterior or posterior operation is impartially discussed, the author summing up as follows: "The question is hardly settled yet, but on the whole the posterior method is to be advised whenever practicable. The extent of the cancer will often determine the surgeon's decision." Special chapters have been inserted dealing with gastric ulcer and choledochotomy.

The chapter on the radical cure of hernia has of necessity been entirely re-written, owing to the great advance in this branch of operative work during the last twenty years and the introduction of Bassini's operation and its modifications. No other operation for radical cure of inguinal hernia is described in detail. The operation of radical cure for femoral hernia has not made any marked advance.

Whitehead's operation for hæmorrhoids is very fully described, the writer stating that he has "performed this operation in some hundreds of cases without a death, without any after bleeding, and without any deep suppuration." The chapter on excision of the rectum has been entirely re-written and brought up to date. The "comment upon the operation of excision of the rectum" at the end of the chapter is well worthy of perusal and careful consideration.

The chapter on excision of the breast has been re-written, considerable advance having been made in these operations during the few years which have elapsed since the publication of the first edition.

There are many new illustrations, and some of the old ones have been discarded.

It would be of considerable advantage if each volume contained its own index. We had hoped to find this altered in the new edition.

